# Forest tree growth is linked to mycorrhizal fungal composition and function across Europe

**DOI:** 10.1038/s41396-021-01159-7

**Published:** 2022-01-10

**Authors:** Mark A. Anthony, Thomas W. Crowther, Sietse van der Linde, Laura M. Suz, Martin I. Bidartondo, Filipa Cox, Marcus Schaub, Pasi Rautio, Marco Ferretti, Lars Vesterdal, Bruno De Vos, Mike Dettwiler, Nadine Eickenscheidt, Andreas Schmitz, Henning Meesenburg, Henning Andreae, Frank Jacob, Hans-Peter Dietrich, Peter Waldner, Arthur Gessler, Beat Frey, Oliver Schramm, Pim van den Bulk, Arjan Hensen, Colin Averill

**Affiliations:** 1 Department of Environmental Systems Science, ETH Zürich, Zürich, Switzerland; 2 Netherlands Food and Consumer Product Safety Authority, National Reference Centre, Wageningen, The Netherlands; 3 Royal Botanic Gardens, Kew, TW9 3DS UK; 4 Department of Life Sciences, Imperial College London, Ascot, SL5 7PY UK; 5 Department of Earth and Environmental Sciences, The University of Manchester, Manchester, M13 9PT UK; 6 Swiss Federal Institute for Forest, Snow and Landscape Research WSL, Birmensdorf, Switzerland; 7 Natural Resources Institute Finland, Rovaniemi, Finland; 8 Department of Geosciences and Natural Resource Management, University of Copenhagen, DK-1958 Frederiksberg C, Denmark; 9 Environment & Climate Unit, Research Institute for Nature and Forest, Geraardsbergen, Belgium; 10 State Agency for Nature, Environment and Consumer Protection of North Rhine-Westphalia, 45657 Recklinghausen, Germany; 11 Thuenen Institut of Forest Ecosystems, 16225 Eberswalde, Germany; 12 Northwest German Forest Research Institute, 37079 Göttingen, Germany; 13 Sachsenforst State Forest, 01796 Pirna OT Graupa, Germany; 14 Bavarian State Institute of Forestry, Freising, D-85354 Germany; 15 The Netherlands Organization for Applied Scientific Research at Petten, 1755LE Petten, The Netherlands

**Keywords:** Microbial ecology, Forest ecology, Fungal ecology, Biogeochemistry

## Abstract

Most trees form symbioses with ectomycorrhizal fungi (EMF) which influence access to growth-limiting soil resources. Mesocosm experiments repeatedly show that EMF species differentially affect plant development, yet whether these effects ripple up to influence the growth of entire forests remains unknown. Here we tested the effects of EMF composition and functional genes relative to variation in well-known drivers of tree growth by combining paired molecular EMF surveys with high-resolution forest inventory data across 15 European countries. We show that EMF composition was linked to a three-fold difference in tree growth rate even when controlling for the primary abiotic drivers of tree growth. Fast tree growth was associated with EMF communities harboring high inorganic but low organic nitrogen acquisition gene proportions and EMF which form contact versus medium-distance fringe exploration types. These findings suggest that EMF composition is a strong bio-indicator of underlying drivers of tree growth and/or that variation of forest EMF communities causes differences in tree growth. While it may be too early to assign causality or directionality, our study is one of the first to link fine-scale variation within a key component of the forest microbiome to ecosystem functioning at a continental scale.

## Introduction

For over a century, ecologists have strived to understand how variation in soil microbial communities affects ecosystem functioning [1–6]. Ectomycorrhizal fungi (EMF) are a key component of the forest soil microbiome, forming symbioses with ~60% of trees on Earth [7, 8]. These fungi aid in early tree establishment and growth [9–15], provide access to otherwise inaccessible soil nitrogen (N) [16], and protect tree seedlings from pathogens [17, 18]. A long history of micro- and mesocosm experiments has demonstrated that different EMF species vary by orders of magnitude in their effects on seedling development [9, 12, 13, 19–25], with potential implications for the conservation and management of specific soil communities to promote tree growth in actual forests [21, 26]. However, it remains unclear whether differences in the composition of EMF meaningfully affects the growth of mature trees and entire forests.

Although it may seem intuitive that differences in EMF composition would lead to variation in the functioning of communities, this area has been intensively debated in the literature [13, 22, 27–33]. It is widely recognized that EMF communities display a considerable degree of functional redundancy [34–36], and that overlapping traits at the aggregate community level might overwhelm species-level differences among communities. It is also possible that the effects of EMF communities are too small to be detected relative to other environmental drivers of tree growth or that any effect of EMF composition is simply reflective of the environmental factors which structure those fungal communities in the first place [37]. Exploring the effects of EMF composition relative to variation in abiotic drivers of tree growth across broad environmental gradients can help address these competing hypotheses and determine if variation in EMF composition has meaningful consequences for forest tree growth.

**Fig. 1:**
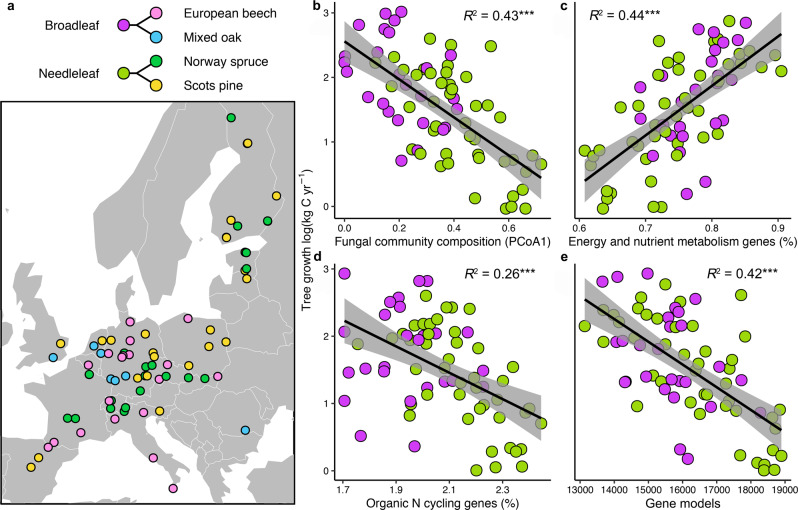
Correlations between the ectomycorrhizal fungal community and tree growth rate across Europe. Map showing the ICP Forests level II study plots with functional tree groups (broadleaf and needleleaf) and dominant tree species (>50% cover) separated by color (**a**). Correlation between tree growth and fungal community composition represented using principle coordinates analysis axis 1 (PCoA1; **b**; see functional tree group colors from panel **a**), fungal energy and nutrient metabolism genes (**c**), fungal organic N cycling genes (i.e. genes encoding for enzymes that EMF produce to access organic N, including peroxidases, multicopper oxidases, peptidases, and proteases (**d**), and the number of gene models identified in the fungal genome as an indicator of metabolic activity (**e**). Fungal energy and nutrient metabolism genes (i.e. ATP production, inorganic N metabolism) are a predefined KEGG metabolic pathway (Pathway 1.2) while organic N cycling genes were aggregated using PFAMs annotations. Gene proportions were calculated as the number of specific gene sequences relative to total gene numbers assigned to operational taxonomic units (OTUs; 97% sequence similarity) weighted based on relative taxon abundance (community weighted mean; CWM). Number of gene models was also calculated as a CWM trait value. Values show predicted tree growth while controlling for the influence of other covariates in the full statistical model (see Materials and Methods). Linear lines, confidence intervals (95%), and *R*^2^ values are displayed, and asterisks indicate significance (*p* ≤ 0.0001).

Until now, our capacity to isolate the effects of EMF composition on tree growth relative to other in situ environmental variation has been precluded by a lack of paired information on forest productivity and EMF composition. Here, we assembled paired ectomycorrhizal community and tree growth data from >13,000 trees across long-term forest monitoring plots in Europe (Fig. 1a) and matched this with molecular fungal taxonomic and functional attributes. This allowed us to explicitly correlate EMF community composition and genomic functional potentials with forest tree growth and to simultaneously examine the influence of EMF community attributes on tree growth while controlling for the potential linear and non-linear effects of climate (mean annual temperature and precipitation), N deposition, soil inorganic N concentrations, and forest stand characteristics (broadleaf vs. needleleaf, stand age, and tree density). Incorporating these environmental predictors is especially important because it allows us to statistically account for well-known drivers of tree growth across the forest network where this work was conducted, notably stand density and age, N deposition, and climate [38]. Previous work has also demonstrated that these environmental variables (plus geographic distance) collectively capture less than 40% of the variation in EMF community composition [39]—which indicates that the EMF and non-fungal predictors of tree growth rate considered here can be disentangled. Lastly, we hierarchically clustered EMF community composition and identified EMF species and growth modalities indicative of communities in slow- versus fast-growing forests.

## Materials and methods

### Data collection and processing

#### Forest inventory data

The International Co-operative Programme on Assessment and Monitoring of Air Pollution Effects on Forests (ICP Forests) has been intensively monitoring several hundred permanent forested plots across Europe since 1995 or later [40]. Each intensive Level II monitoring plot is at least 0.25 ha, and most trees with at least 5 cm diameter at breast height (DBH) are identifiable by a unique number used to make periodic measurements [41]. DBH was measured using a caliper or measuring tape approximately every five years, a commonly used interval for estimating forest growth and yield. Tree species identity is reported for every measured tree, and each plot was classified by the dominant tree species (>50% abundance). We used data from plots dominated by *Pinus sylvestris* (Scots pine), *Fagus sylvatica* (European beech), *Picea abies* (Norway spruce), and *Quercus robur* and *Q. petraea* (pedunculate and sessile oak; hereafter: mixed oak).

We used periodic DBH observations to calculate a diameter increment growth rate for each tree. We removed dead trees, trees with DBH < 5 cm, and trees which shrank over the growth period which were occasionally included in the census. To investigate long-term drivers of tree growth and avoid potential short-term abnormalities, we used the first and last periodic growth measurement to calculate diameter growth increment. The entire period covered was 1999–2017, the mean initial census year was 2005, the mean final census year was 2008, and the mean growth interval was 5.5 years. Although there is some variation in the annual sampling of tree growth versus the fungal community at some sites, previous work has shown that year-to-year variation in fungal communities is low at regional to continental scales [42, 43]. Allometric equations for dominant tree species within our observed size and geographic range were used to calculate aboveground biomass using equations from the GlobeAllomeTree database [44]:

*Pinus sylvestris*12.91-2.8035*(DBH) + 0.3535*(DBH)^2^

*Fagus sylvatica*0.19465*(DBH)^2.418775^

*Picea abies*0.4626*(DBH)^2.133^

*Quercus robur* & *Q. petraea*0.23095*(DBH)^2.27265^

The percentage of tree mass which is carbon versus other elements was estimated using IPCC Good Practice Guidance for Land Use, Land-Use Change and Forestry and assumed to be 50% [45].

We also downloaded metadata for each plot (where available) within the ICP Forests database based on long-term in situ measurements using harmonized methods for N deposition (similar to ref. [46]), soil pH, and soil inorganic N concentrations (ammonium and nitrate). Because N deposition measurements were incomplete across the study plots, we used N deposition data from 2010–2015 from the European Monitoring and Evaluation Program (EMEP) at a 1-km spatial resolution, which was tightly correlated with the ICP Forest N deposition measurements (*R*^2^ = 0.5, *p* < 0.0001; similar to ref. [38]). Soil solution measurements for inorganic N were made on soil water fractions collected weekly, bi-weekly, or monthly except at sites where water for collection was too scarce and/or where snow and ice prevented winter sampling. Soil pH was measured in CaCl_2_ using potentiometry. We calculated inorganic N and soil pH values obtained between 2010–2017 at a 0–25 cm depth. This falls within the same time-frame and soil depth as the EMF community sampling. Detailed description of analytical methods may be found in the ICP forest manual [47]. We also downloaded and used information on the average stand age class of each plot. Lastly, we downloaded 1-km spatial resolution mean annual temperature (MAT) and precipitation (MAP) data from WorldClim2 [48].

#### Fungal ITS data analysis

Full length ITS DNA sequences obtained from ectomycorrhizae by [39] were used for the EMF community analysis. Complete information on the sampling is described in the original publication and in ref. [49]. In short, ectomycorrhizae were characterized for 137 ICP Forest level II sites between 2006–2008 and 2013–2015. At each site, four soil cores (25 cm deep, 2 cm diameter) were collected from underneath 24 tree-to-tree transects and sampled for 288 ectomycorrhizae per plot. A total of 87 plots had paired EMF community and forest inventory data and 71 plots had complete data for all variables included in the full statistical models.

After downloading the DNA sequence data from DRYAD, a fastq file was produced using Phred scores from the qual file using the Unipro UGENE software [50], and sequences were trimmed at a Phred score threshold of <20 and sequences <100 bp were removed using the Sequence quality trimmer function in UGENE. Of the 35,989 sequences, we retained 32,219 after quality control. We then used the usearch_global function in usearch (v11) [51] to match sequences at a 97% sequence similarity threshold against the full UNITE + INSD database (2018-11-18) [52].

Using the fungal genomic portal, MycoCosm [53], we assigned ITS sequences genomic traits related to the functioning of ectomycorrhizae. Similar methods have been developed for phylogenetic inference using prokaryote 16S surveys [54, 55] but were modified for ITS analysis in this study since the ITS region is less suitable for phylogenetic analyses across all fungi. We used the MycoCosm All-Fungi Species Tree (downloaded from [56] to determine whether there was phylogenetic signal to the genomics traits of interest, including numbers of enzyme nomenclature (EC) related protein sequences (hereafter: gene numbers) based on functions in the Kyoto Encyclopedia of Genes and Genomes (KEGG) and protein family (PFAM) groups related N cycling (i.e., N permeases and ammonium sensing genes), proteolysis (i.e., peptidases, proteases), decomposition of N-bearing compounds (i.e., oxidases and multicopper oxidases), and fungal cell wall biosynthesis (i.e., chitin and glucan biosynthesis). We also summed peptidase, protease, and decomposition of N bearing compounds to study total organic N cycling genes given the overall importance of ectomycorrhizal organic N acquisition in forest ecosystems [16]. Many KEGG functions are directly relevant to the exchanges occurring between host plants and EMF, including energy and nutrient, nitrogen and amino acid, and carbohydrate metabolism and various anabolic and catabolic pathways. We also considered the total number of gene models per genome as a proxy for metabolic activity [57] and specifically how microorganisms grow and metabolize carbon.

The phylogenetic tree was pruned to only include species from genera in our dataset which restricted the analysis to species from 101 fungal genera. We tested for phylogenetic signal of gene numbers using the phylosig function in the phytools package [58] using 10,000 simulations and after setting method = “K” and method = “lambda” to calculate Bloomberg’s K and Pagel’s lambda, respectively (see Table S1). After screening for phylogenetic signal, we first assigned functional genes to direct species matches, accounting for 50% of the matched OTUs, and then to species and genera without direct reference genome matches when there was significant phylogenetic signal. Where there was phylogenetic signal, but not a direct species match, we assigned the average genus-level gene number from all species within a genus in MycoCosm to an OTU. OTUs were not included in this analysis if they were not assigned a genus-level taxonomy. See Fig. S1 for a decision tree outlining this approach. To account for differences in genome size which could lead to spurious correlations, we standardized for genome size by calculating the proportion of genes representative of each function relative to genome size using the total number of genes, an approach similar to ref. [56]. Proportional gene numbers were then weighted based on relative abundance in the OTU table to calculate community weighted mean (CWM) trait values. Trait values were weighted based on relative abundance using the base weighted.mean R function. Of the 101 genera identified in the dataset, 54 KEGG and 47 PFAM assignments were made; of the 1022 OTUs in the dataset, 512 KEGG and 455 PFAM assignments were made, and of the total sequences, 46% were assigned KEGG and 25% PFAM annotations. Half (258) of the assigned OTUs were exact reference species matches.

It is important to address that inferring potential microbiome functions from DNA metabarcoding studies is very common [55, 59–62] but has been debated in the literature [63]. Criticism has focused on 16S analyses inferring functional profiles from environmental samples when there is poor overlap between observed taxa and those with reference genomes [63]. Yet in our study, we largely avoided this issue by focusing on root-associated EMF—50% of the species and genera identified in our dataset have a direct species or genus-level match to reference genomes in MycoCosm (see above). This method is also uniquely informative. We could not use metagenomics since high bacterial ribosomal copy numbers largely prevents fungal analyses [64], and alternative DNA-based methods are laborious and cost-prohibitive [65]. Thus, cautiously assigning functional potentials to EMF on a study-by-study basis using reference genomes may be a viable technique as long as there is high overlap between observed and reference taxa in MycoCosm.

### Data analysis

#### Fungal community analyses

All statistical analyses were conducted in R (v3.6.1) [66], and significance was set to *p* ≤ 0.05. We calculated beta diversity, fungal richness (# of OTUs), community diversity (Shannon index), and CWM functional gene values. First, we randomly rarified the dataset to the lowest number of observations (115 DNA sequences per plot) using the rrarefy function in the vegan package [67]. This is a robust sequencing depth for EMF Sanger sequencing [68–70], and has previously been shown to correspond well with high-throughput EMF DNA sequencing techniques [71, 72]. We then calculated relative OTU abundances, produced a Bray–Curtis dissimilarity matrix using the vegdist function (vegan), and represented EMF composition using principal coordinates analysis (PCoA) via the pcoa function in the ape package [73]. Fungal richness and diversity (Shannon index) were calculated using the specnumber and diversity functions in vegan, respectively. The relationship between fungal community composition, fungal functional potentials inferred using CWM gene numbers, and tree growth was assessed using distance-based redundancy analysis (db-RDA).

Using the OTU-based Bray–Curtis dissimilarity matrix (computed for all 137 sites), we performed hierarchical clustering using the base hclust function in R. The optimal number of clusters was evaluated using the elbow method [74]. We then used analysis of means and the ANOM function in the ANOM package [75] to identify clusters from sites with greater and lower tree growth rates than the overall mean. This resulted in a fairly balanced number of sites between slow and fast growth communities in the needle- (*n*_slow_ = 14 vs. *n*_fast_ = 12) and broadleaf (*n*_slow_ = 14 vs. *n*_fast_ = 19) sites, respectively. EMF cluster was then used as a discriminatory factor for indicator species analysis performed using the multipatt function in the indicspecies package [76].

#### Tree growth models using generalized additive models

Generalized additive models (GAMs) were used to predict tree growth (kg C yr^−1^) rates at the plot level using the gam function in the mgcv package [77]. We used statistically independent fixed effects (*r*^*2*^ < 0.5) including tree density, forest stand age class, a binary categorical factor for needleleaf and broadleaf tree types, MAT, MAP, N deposition, soil pH, and inorganic N concentrations (soil ammonium + nitrate concentrations). We fit smoothing functions using penalized regression splines to reduce over-fitting to predictors with non-linear correlations to tree-growth, including stand age and stand density (Fig. S2). Spline fits were assessed using the plot.gam function, and smoothness selection optimization and basis dimensions were determined using the gam.check function. We used restricted maximum likelihood methods for smoothing parameter estimation. Separate models were created for each fungal parameter without smoothing functions. Models were inspected for normal distribution of the residuals, residual versus fitted plots, and issues of multicollinearity among predictors based on variance inflation factors. Growth was natural log transformed to satisfy the assumption of homoscedasticity.

We also estimated plot level tree growth (Mg C ha^−1^ y^−1^) rates as opposed to individual tree growth rates (kg C y^−1^) to explore differences at the stand level between forests classified as part of the slow- versus fast-tree growth associated EMF community types. Since periodic DBH measurements are not made on every tree in the plots (i.e., only a subset of trees in level II ICP Forest plots are measured for growth) we could not simply compute the sum biomass C gain of all trees. We therefore randomly sampled with replacement trees which are periodically measured until reaching the in situ stem density of each plot 1000 times. From this distribution, we summed the biomass-C gain across all trees. Following the same procedure as above, we then modeled plot level tree growth using fungal community composition as a predictor variable. Significant differences between sites classified as part of the slow- versus fast-tree growth associated EMF community types were evaluated using heteroscedastic *t*-tests.

For visualization, we calculated model partial residuals with respect to the fungal predictor [78], and then added the effect of all other predictors at their mean values, so that data could be interpreted on their original scale. Mathematically, this can be expressed as:

$$y\_i = f(x\_i {\,}^{\wedge} 1) + f(x^{-} {\,}^{\wedge} (2\backslash - n)) + \varepsilon$$

Where $y\_i$ is the vector of partial residuals on the original scale of the data, $x\_i \wedge 1$ is the vector of observed values with which partial residuals are calculated relative to, $x - \wedge (2\backslash - n)$ are the remaining model covariates at their mean values, and $\varepsilon$ is the vector of fitted model residuals. $f(x)$ represents the fitted functional forms of how each independent variable affects the dependent variable output by the generalized additive model.

## Results

### Trends in tree growth rate and co-variable quality and independence

Correlations between non-fungal environmental predictor variables and tree growth rate were consistent with expectations based on previous studies from the ICP Forest network. Mean annual temperature (*r* = 0.39, *p* < 0.0001), stand density (*r* = −0.38, *p* < 0.0001), and N deposition (*r* = 0.27, *p* < 0.01; see Fig. S2) were most tightly correlated with tree growth. These were also the top three predictors of tree growth rate in a recent study conducted across ~300 ICP level II plots [38]. Tree growth rate also varied non-linearly with stand age class, an expected pattern in boreal and temperate forests [79] and across the ICP Forest network [38]. Inorganic N concentrations and soil pH were both positively correlated with tree growth rate, but this was not significant. Consistent with previous studies [39], environmental predictor variables were not highly correlated with the fungal community, and their individual effects were distinguishable, as indicated by low variance inflation factors in the full statistical models [80] (1.3-2.6; Table S2). Expected patterns between tree growth rate and non-fungal environmental predictor variables and sufficient independence among non-fungal predictors and the fungal community supports the idea that we can address our main research objectives to explore the relative effects of EMF community variation on tree growth rates.

### Fungal community and functional gene linkages to tree growth rate

Multiple features of the EMF community were linked to tree growth rates and were statistically independent of other environmental drivers of tree growth. Tree growth was strongly correlated with fungal community composition (represented as PCoA axis 1; Fig. 1b), and explained more variation than mean annual temperature and precipitation, N deposition, soil inorganic N concentrations, and soil pH (see variance partitioning results in Fig. S3). The total model explained 54% of the variation in tree growth rate. The same model, but without the fungal community predictor, captured 37% of the variation. Similar fungal composition effects were also observed when tree species were examined individually (Fig. S4). Conversely, fungal richness (*p* = 0.89) and diversity (Shannon index; *p* = 0.57) were not significantly correlated with tree growth (Table S3). These results suggest that EMF alpha diversity is not strongly linked to tree growth while variation in EMF community composition (beta diversity) is a top predictor of tree growth rate across Europe.

To examine which functional features of the fungal community were associated with changes in tree growth rates, we evaluated functional genes related to nitrogen acquisition, soil organic matter decomposition, and fungal growth as community-weighted functional gene proportions. Among all gene groups, fungal energy and nutrient metabolism gene proportions were the strongest predictors of tree growth (Fig. 1c). These metabolic pathways reflect genomic investments in releasing chemical energy, including ATP production (e.g., oxidative phosphorylation, dissimilatory sulfate reduction) and inorganic N metabolism (i.e., N reduction, oxidation, transport). Conversely, the relative abundance of organic N cycling genes, including peptidases, proteases, multicopper oxidases, and peroxidases was negatively correlated with tree growth rate (Fig. 1d). No genes related to fungal growth were significantly correlated with tree growth rate (e.g., glycan biosynthesis, *p* = 0.11; glucan biosynthesis, *p* = 0.67; chitin biosynthesis, *p* = 0.11; Table S3), but the number of gene models per genome was negatively correlated with tree growth (Fig. 1e). These results show that fungal communities with fewer genes in their genome and which are energetically more active and inorganic N specialized are linked to fast tree growth.

**Fig. 2:**
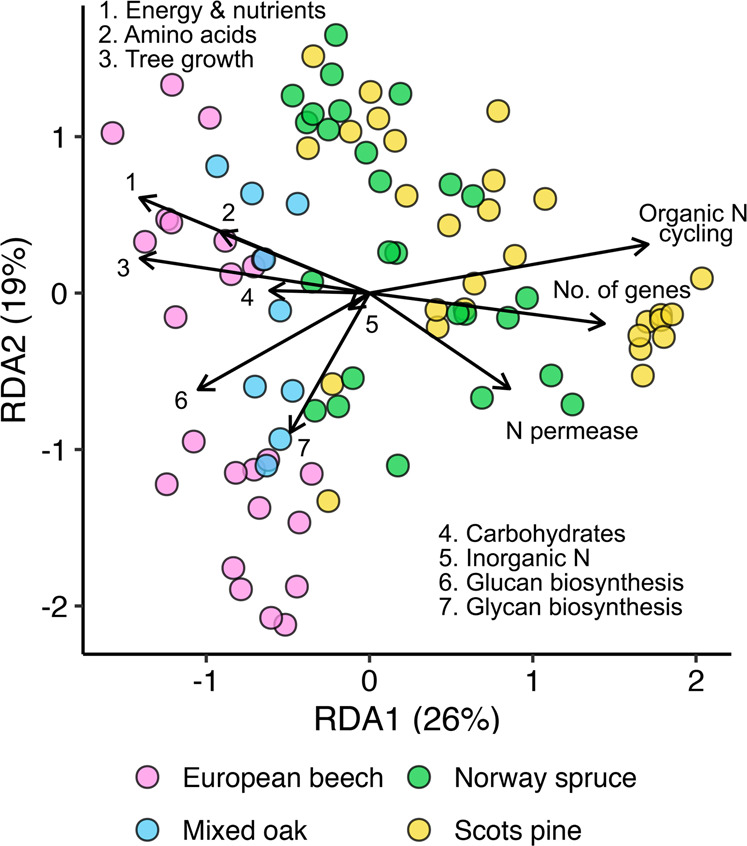
Fungal composition depicting community types and genomic functional gene potentials associated with tree growth rate. Distance-based redundancy analysis (RDA) performed using fungal relative abundances converted to Bray–Curtis dissimilarities and fungal functional gene community weighted mean proportions and tree growth rate as explanatory variables. Note the configuration of fungal communities associated with fast tree growth corresponds with higher proportions of energy and nutrient metabolism, amino acid metabolism (AA), carbohydrate metabolism, and to a lesser extent, inorganic N metabolism genes, glucan biosynthesis (Glucan), and glycan biosynthesis (Glycan) genes. Fungal communities associated with slow tree growth correspond with organic N cycling (peptidases, proteases, multicopper oxidases, peroxidases), N permease gene proportions, and number of gene models in the genome (No. of genes).

To better understand differences in functional capacities between fungal communities linked to slow- vs. fast-tree growth, we examined differences in functional gene composition among EMF community types using distance-based redundancy analysis. Consistent with our full statistical growth models, fast-tree growth associated EMF communities harbored higher proportions of energy and nutrient metabolism genes, lower proportions of organic nitrogen cycling and N permease genes, and had fewer gene numbers in their genomes (Fig. 2). While these differences were observed in both broad- and needleleaf dominant forests, needleleaf inhabiting EMF communities had lower proportions of energy and nutrient metabolism genes, higher proportions of organic N cycling genes, and a greater total number of gene models compared to broadleaf forests.

**Fig. 3:**
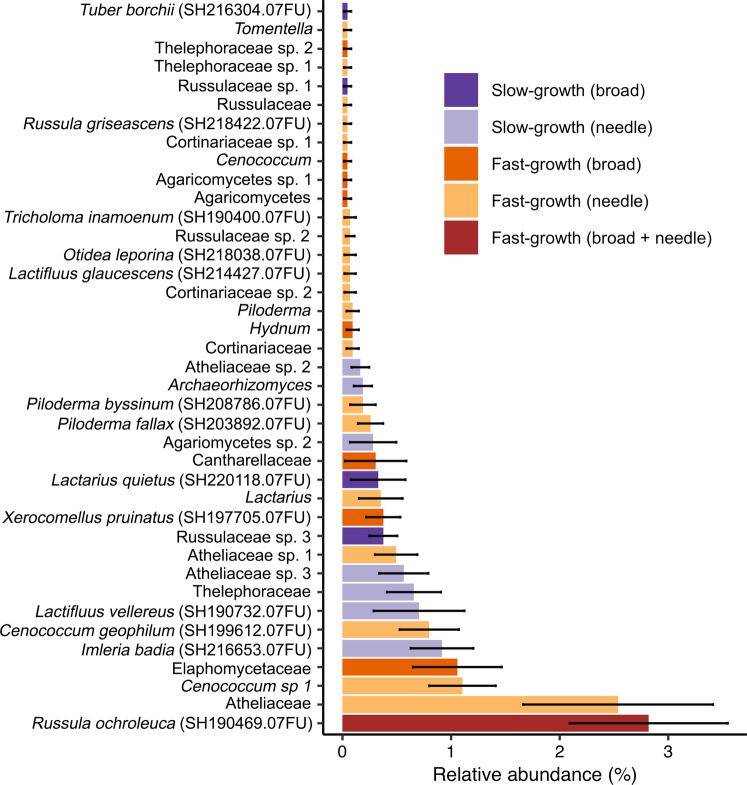
Fungal taxonomic indicator species representative of the slow- and fast-tree growth associated EMF community clusters in needleleaf (Scots pine and Norway spruce) and broadleaf (European beech and mixed oak) forests. The relative abundance of significant fungal indicator taxa identified to the highest taxonomic level and organized for visual purposes by rank abundance. Each species-level taxon is annotated with a reference species hypothesis identifiable by the internal transcribed spacer region DNA sequence aligned at ≥97% sequence similarity to references in the UNITE database. Bars show the mean relative abundance of taxa across all plots where taxa occurred, and error bars show the standard error.

Next, we identified fungal taxa that were indicative of the hierarchically clustered slow- and fast-tree growth associated EMF community groupings (Fig. 3). Forty operational taxonomic units (OTUs) were significant indicator species of the slow- (11 OTUs) and fast-tree growth (29 OTUs) associated EMF communities (Table S4). *Russula ochroleuca* was the only indicator species of fast tree growth in both the broad- and needleleaf dominant stands. Other taxa were strictly indicators of needle vs. broadleaf forests. For example, two *Cenococcum* OTUs, including *C. geophilum* were indicators of fast needleleaf tree growth, while a distinctive *Cenococcum* OTU was indicative of fast broadleaf tree growth. A suite of less-common *Piloderma* OTUs, including *P. fallax* and *P. byssinum*, were indicators of fast-tree growth in the needleleaf stands but not the broadleaf stands. Fungal OTUs identified at the family-level included indicators of both slow- and fast-tree growth.

**Fig. 4:**
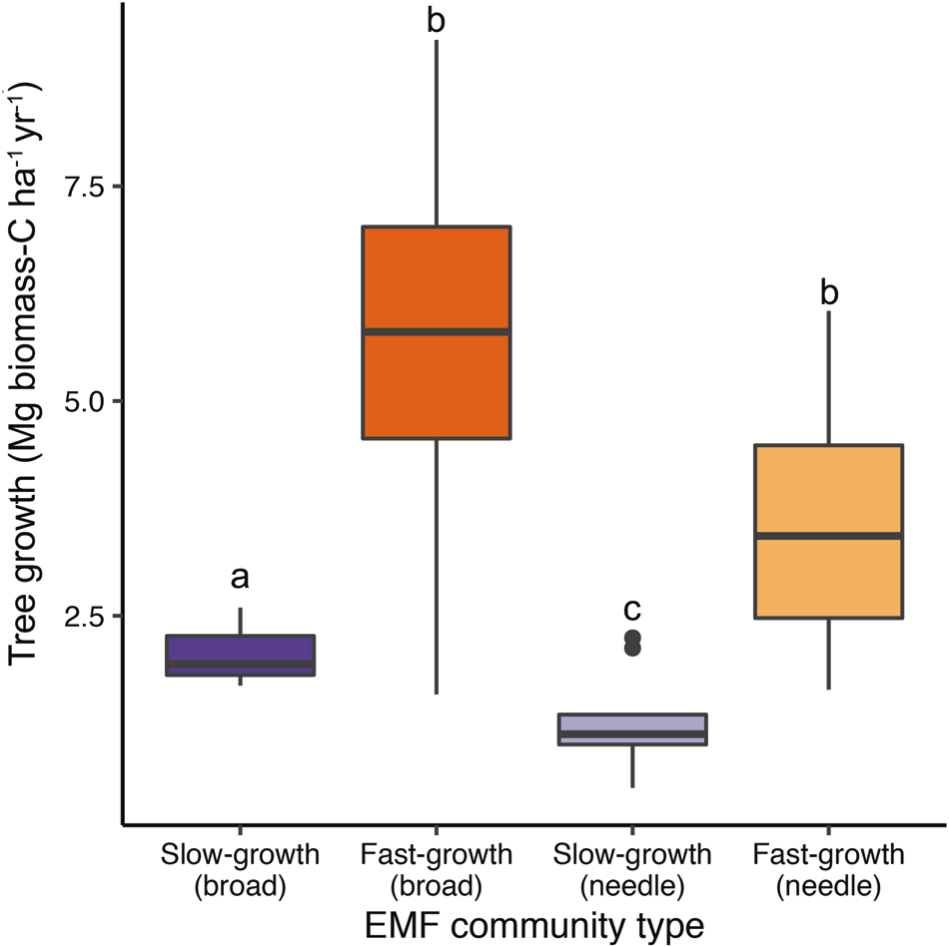
Annual aboveground forest tree growth aggregated at the stand level comparing forests classified as part of the slow- and fast-tree growth associated ectomycorrhizal fungal community types in needleleaf (Scots pine and Norway spruce) and broadleaf (European beech and mixed oak) forests. Values show predicted tree growth rates at sites classified as part of the slow vs. fast tree growth associated EMF communities while controlling for the influence of other covariates. Significantly different values were evaluated using heteroscedastic *t*-tests. Different lowercase letters indicate significant differences (*p* ≤ 0.05).

At the stand versus individual tree level, we compared growth rates between all forests classified as part of the slow- or fast-tree growth associated EMF community groupings after controlling for the environment and other covariates. Stand-level tree growth rate was 2.3 and 5.9 Mg C ha^−1^ yr^−1^ in the slow- versus fast-tree growth associated broadleaf EMF community forests, respectively, and it was 1.1 and 3.0 Mg C ha^−1^ yr^−1^ in the slow- versus fast-growth associated needleleaf EMF community forests, respectively (Fig. 4). This approximate tripling of tree growth rates among forests with particular ‘fast-tree growth’ communities is equivalent to the variation in tree growth driven by other large-scale environmental predictors in this analysis, including mean annual temperature and precipitation.

## Discussion

The importance of EMF in forest ecosystems is of long-standing interest [3, 4, 81], but more recently, focus has shifted towards understanding the implications of fine-scale EMF community variation for ecosystem function [5, 33, 82]. A central forest function affected by EMF is tree growth, but the effects of ectomycorrhizal community variation on tree growth in actual forests have not been well studied, until now, due to an absence of paired molecular mycorrhizal and high resolution forest inventory data. Here, we show that, along with climate, soil, and stand characteristics, the composition of soil EMF communities is a prominent predictor of forest growth across the European continent. These findings bring clarity to the debate around whether microbial species level differences impact microbial functions. A common argument against species level effects is that fungal communities are characterized by high levels of functional redundancy [34–36]. However, we show that compositional and functional differences at the aggregate community level overwhelm species trait overlap and are linked to a three-fold difference in tree growth rate (Fig. 1b). While our results are based on observational analyses and it is not yet possible to assign causality or directionality to the correlations identified here (i.e., is tree growth correlated with variation in EMF communities and/or is EMF community variation driven by tree growth?), our study is the first to link fine-scale EMF community variation to ecosystem functioning at a continental scale.

### Ectomycorrhizal composition is linked to tree growth rate via N cycling potentials and genome characteristics indicative of mycorrhizal exploration types

Tree growth rate was strongly correlated with variation in fungal community composition. While tree growth was positively correlated with fungal richness and diversity (alpha diversity), these effects were not significant. This lends support to the idea that EMF identity, and to a lesser extent alpha diversity, are drivers of tree nutrition and growth [23, 24, 83–85]. Why EMF richness was not correlated with tree growth may be due, in part, to high EMF richness levels. Diversity-function relationships saturate when tree richness is high [86], and EMF richness vastly exceeds that of plants. Further and because there is mixed evidence for negative [87] and positive [88–90] EMF alpha diversity effects on tree seedling development, EMF alpha diversity effects are likely context dependent (i.e., in low diversity EMF systems) and localized [83]. Conversely, EMF composition effects may be widespread throughout actual forests and are predictable at large spatial scales. This idea has been insinuated for decades based on experimental pairings between tree seedlings and EMF from diverse clades and functional groups [9, 12, 19–21, 23, 24], but until now, it has not been tested across actual forests with large variation in tree growth rates nor has it been studied in the context of variation in fungal functional genes. We specifically find that variation in fungal composition is linked to contrasting nutrient acquisition strategies and that slow tree growth is associated with EMF communities harboring high organic N cycling gene proportions. Our findings hold significant implications for fungal conservation (i.e., monitoring of taxa which promote forest tree growth) and responses to global changes (i.e., if climate change selects for different EMF functional groups) that could ripple up to affect forest productivity.

By analyzing the functional potential of EMF communities, we also identified two contrasting functional axes correlated with tree growth related to inorganic vs. organic N acquisition. Ectomycorrhizal fungi have varying capacities to take up inorganic [88, 91] and organic [92] N sources. Here, we found a positive effect of fungal energy and nutrient metabolism genes, including inorganic N metabolism gene proportions, on tree growth rate. Since EMF largely function to provide host plants with N [16], this finding is consistent with fundamental mycorrhizal theory. However, we also found a negative effect of organic N cycling genes on tree growth rate, including oxidative enzymes, peptidases, and proteases. This effect was strongly driven by proportions of multicopper oxidases which were most negatively correlated with tree growth (*p* < 0.0001; Table S3). These enzymes depolymerize soil organic matter, one of the major rate limiting steps in making N bioavailable [93]. These enzymes are also energetically expensive microbial investments [94–96]. The net cost to plants imposed by EMF may be high when partnered primarily with organic N specialized communities if they require high host plant carbon allocation [97, 98]. Of course, organic N specialized communities may be selected for by low inorganic N availability [96]. However, the proportion of organic N cycling genes in our study was weakly and significantly positively correlated with inorganic N availability (Fig. S5). This suggests that communities with higher organic N acquisition potential are not responding to inorganic N limitations and might even make it more available. Importantly, gene numbers and their associated processes are not always correlated [99], highlighting the importance of these values as potential genomic functions. Our functional gene analysis also has drawbacks due to reliance on databases. Assignments were not possible if OTUs did not have a species or genus-level taxonomic assignment in the UNITE database (41% of the OTUs lacked a genus/species assignment) nor if species or genera were absent from MycoCosm (50% of the OTUs were absent). Even though we could not create a complete functional representation of the community, we were still able to observe strong N cycling gene correlations with tree growth, albeit only for part of the EMF community.

**Fig. 5:**
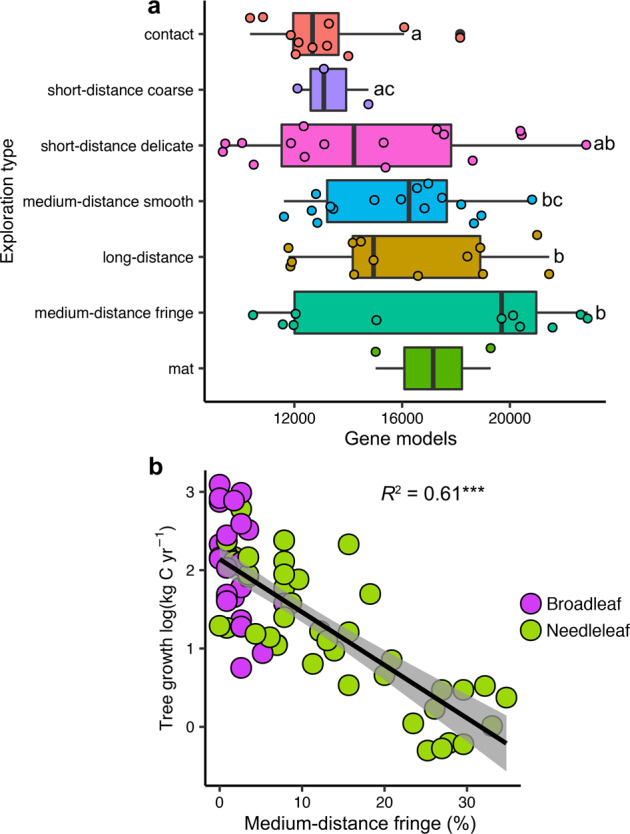
The relationship between ectomycorrhizal fungal exploration type, number of gene models, and tree growth rate. Number of gene models was summarized by ectomycorrhizal exploration types based on species and genera identified in this study (**a**). Examples of genera with particular exploration types in the study are included in parentheses. Significant differences were tested using heteroscedastic *t*-tests to account for unequal sample numbers and differences are indicated using different lower-case letters (*p* ≤ 0.05). No comparisons were made against the mat-forming type as there were too few species with sequenced genomes identified in our study. The correlation between tree growth rate and the relative abundance of medium-distance fringe type EMF (**b**). Values show predicted tree growth while controlling for the influence of other covariates in the full statistical model. Linear lines, confidence intervals (95%), and *R*^2^ values are displayed (*** = *p* ≤ 0.0001). Note that this correlation was significant for both broad- (*R*^2^ = 0.18, *p* = 0.03) and needleleaf (*R*^2^ = 0.66, *p* < 0.0001) forests when examined individually.

In addition to variation in N acquisition potentials, fungi exhibit variable size and complexity to their genomes with implications for overall metabolic function. We found a negative correlation between tree growth rate and the number of gene models per genome. To explore this further, we linked EMF species found in our dataset to their exploration types using the Fungal Traits database (v1.2; see Table S5 for a summary of the exploration type assignments) [100]. Medium- and long-distance soil exploration fungi harbor higher numbers of gene models compared to contact and short-distance coarse types (Fig. 5a). Medium and long exploration types may require more C from their host plants [101], a C cost that could constrain tree growth [98]. The relative abundance of medium-distance fringe EMF, the group with the largest number of gene models and which produce the most extensively fanning hyphae and rhizomorphs [102], was negatively correlated with tree growth rate (Fig. 5b), particularly in needleleaf stands where they were at higher relative abundance than broadleaf stands. Conversely, the relative abundance of contact type EMF, which produce the least emanating hyphae, was positively correlated with tree growth rate (*R*^2^ = 0.11, *p* = 0.005) and this effect was the same between forest types (Fig. S6). Thus, proportions of different EMF soil exploration types in communities may be an underlying mechanism to explain variation in tree growth as they have also been linked to a tipping point in tree mineral nutrition in the same forests [103]. How this may apply to older forests with different proportions of EMF exploration types remains uncertain. EMF which produce high amounts of emanating hyphae may peak at intermediate forest ages [104], including in our analysis at 90 years old (Fig. S7) and may shift towards greater organic N usage with stand age [105]. It thus remains premature to assign causality among tree growth rates, EMF community gene numbers, and exploration types or to disentangle the directionality of these correlations across time, but this is an exciting new area of molecular mycorrhizal research with implications for forest tree growth.

### Fungal taxa associated with slow- and fast-growing forests

A number of the commonly observed slow- and fast-tree growth associated indicator species have well-described N metabolism strategies which could affect tree growth. Notably, *Russula ochroleuca*, the most common indicator of fast broad- and needleleaf tree growth, is known to forms symbioses with both tree types [39] and has been previously classified as nitrophilic, being at increased relative abundance where N deposition levels are high [97]. This fungus takes amino acids up more slowly than other common European EMF and may primarily use inorganic N [92], which is energetically favorable compared to organic N [94, 106] and could promote tree growth. Other indicators were linked to tree growth rates consistent with previously described effects. For example, the fast-tree growth associated taxon in needleleaf forests, *Cenoccocum geophilum*, has been experimentally shown to boost *Pinus tabulaeformis* growth [107], in addition to numerous other tree species as it a host-generalist [9, 108], and it provides a range of benefits to host plants under stressful conditions [11, 109]. *Piloderma* species were also indicators of fast-tree growth in the needleleaf stands, and *Piloderma* has been shown to positively correlate with Scots pine gross primary productivity in Finland [110]. For some taxa, host specificity may explain ‘slow-growth’ designations. For example, *Lactarius quietus* was indicative of slow needleleaf tree growth, potentially because it is a broadleaf specialist [39]. Observations that a number of indicator species identified here are associated with tree growth rates consistent with previous studies lends further support to our findings.

Despite our results being consistent with decades of mesocosm studies and multiple different angles of mycorrhizal theory on N cycling and C demand by different soil exploration types, we cannot discern whether observed fungal effects drive and/or respond to variation in tree growth rates—a limitation of any large scale observational study. The fungal effects observed in this study likely emerge as the result of a combination of positive and negative feedbacks and additional environmental attributes. It is additionally possible that the fungal effects we observed represent environmental factors not considered in our study—i.e., our fungal effects are ‘merely’ powerful bio-indicators of unknown, underlying drivers of tree growth. However, by including the most important drivers of tree growth rate across the ICP Forests network in our full statistical models, including N deposition, climate, and stand characteristics (*sensu* [38]), we can at least confirm that these fungal effects capture unique variation in tree growth above and beyond the most widely recognized environmental drivers of tree growth. While EMF fungal communities are also affected by many of the environmental factors considered here, climate, N deposition, soil properties, host plant characteristics, and geography only capture 37% of the variation in EMF community composition in this dataset [39] and even less variation in other studies [111]. This, in addition to low variance inflation factors in our statistical models, indicates that observed fungal effects are not simply reflections of the main environmental drivers of tree growth across the ICP Forests network. Nevertheless, future experimental work that manipulates actual forest EMF communities will be essential to validate and tease apart the directionality of our findings.

### Stand-level tree growth rates are tripled in association with fast-growth communities

Our results suggest that EMF community differences strongly impact mature tree growth, and that this could have important impacts on forest carbon storage. To test this, we aggregated individual tree-level growth data to the plot level and compared all forests classified as part of the slow- or fast-tree growth associated EMF community groups after controlling for the environment and other covariates. Fungal communities associated with fast tree growth were linked to an approximate tripling in tree growth (Fig. 4). While these differences are large, and at the high ranges for these trees [112], they do not consider how tree growth rates may covary with tree mortality rates, belowground productivity, or soil carbon cycling. We emphasize that for these reasons, observed differences in tree-growth do not necessarily translate to changes in ecosystem-scale carbon storage. Nevertheless, tree growth rate is a fundamental component of the forest carbon cycle, and our study is the first to describe how fine-scale variation within a key group of the forest microbiome may control forest productivity at a vast spatial extent.

In conclusion, forests are one of the largest terrestrial C sinks. Understanding the mechanisms underpinning the strength of this forest C sink is critical for projecting land C storage under current and future climate scenarios. Here, we show that, along with climate, soil, and stand characteristics, the composition of EMF communities may be a prominent factor governing forest tree growth across the European continent. This is consistent with decades of micro- and mesocosm studies suggesting that EMF community composition regulates the development of individual tree seedlings. Our study highlights a division where fast growing forests harbor inorganic N specialized communities with high proportions of contact type ectomycorrhizae while slow growing forests are enriched in organic N EMF specialists dominated by medium-distance fringe soil exploration types. These continental patterns provide initial insights into integrating mycorrhizal fungal traits (i.e., N cycling genes studied here) into plant-growth models, such as FUN [113], and the potential for managing forest soil EMF communities to regulate forest tree growth rate, analogous to large scale epidemiological work which links variation in human gut microbial community composition to human health [114, 115]. Specifically, the conservation of fungi which regulate tree growth rates and the development of fungal inoculations targeted to specific forest functions (i.e., tree growth rates and/or nutrient cycling) are among two emergent opportunities for managing forests to promote forest C storage under a changing climate.

## Supplementary information

Updated supplement

## Data Availability

All data used to analyze the fungal community is available from the original authors via DRYAD (https://datadryad.org/) with, 10.5061/dryad.cr70qc8. Access to the forest inventory and environmental data sets is available via the ICP Forests network upon request (http://icp-forests.net/page/data-requests). Restrictions apply to the availability of these data without a formal data request, though may be made available for the purposes of replicating this analysis. R scripts for working up the raw data and analysis are available at https://gitlab.ethz.ch/manthony/icp-forest-growth-and-ecm-fungi.
