# Supplementary material for: Forest tree growth is linked to mycorrhizal fungal composition and function across Europe
Source: ISME J. 2022 Jan 10;16(5):1327–36. doi: 10.1038/s41396-021-01159-7 (PMC9038731; doi:10.1038/s41396-021-01159-7)
Supplement: Supplementary file 1 — Updated supplement [file 41396_2021_1159_MOESM1_ESM.docx]

**Supplementary Information**

**Forest tree growth is linked to mycorrhizal fungal composition and function across Europe**

Mark A. Anthony^1,†^, Thomas W. Crowther^1^, Sietse van der Linde^2^, Laura M. Suz^3^_,_ Martin I. Bidartondo^3,4^, Filipa Cox^5^, Marcus Schaub^6^, Pasi Rautio^7^, Marco Ferretti^6^, Lars Vesterdal^8^, Bruno De Vos^9^, Mike Dettwiler^1^, Nadine Eickenscheidt^10^, Andreas Schmitz^10, 11^, Henning Meesenburg^12^, Henning Andreae^13^, Frank Jacob^13^, Hans-Peter Dietrich^14^, Peter Waldner^6,^ Arthur Gessler^1,6^, Beat Frey^6^, Oliver Schramm^6^, Pim van den Bulk^15^, Arjan Hensen^15^, Colin Averill^1^

Correspondence should be addressed to Mark Anthony ([mark.anthony@usys.ethz.ch](mailto:mark.anthony@usys.ethz.ch))

**Table S1**. Analysis of phylogenetic signal in the number of genes encoding for core KEGG metabolic pathways and PFAM genes associated with organic N metabolism, decomposition, and fungal cell wall biosynthesis. We computed BOTH Pagel’s lambda and Bloomberg’s *K*. KEGG and PFAM groups without phylogenetic signal are marked using red font. The phylogenetic tree is based on multiple alignment of proteins shared in most of MycoCosm fungi and is changing upon addition of new genomes.

|  |  | Lambda | *K* |
| --- | --- | --- | --- |
| **KEGG metabolic path** | Amino acid metabolism | 0.98 | 0.06 |
|  | Energy and nutrient metabolism | 0.6 | 0.006 |
|  | *Nitrogen metabolism* | 0.45 | 0.03 |
|  | *Sulfur metabolism* | 0.56 | 0.005 |
|  | *Oxidative phosphorylation* | 0.17 | 0.002 |
|  | Carbohydrate metabolism | 0.88 | 0.01 |
|  | *Starch & sucrose metabolism* | 0.98 | 0.04 |
|  | *Pentose phosphate pathway* | <0.01 | 0.002 |
|  | *Glycolysis* | 0.86 | 0.18 |
|  | *Fructose and mannose metabolism* | 0.89 | 0.11 |
|  | Biosynthesis of polyketides and non-ribosomal peptides | 0.91 | 0.007 |
|  | Biosynthesis of cofactors & vitamins | 0.94 | 0.02 |
|  | Lipid metabolism | 0.96 | 0.04 |
|  | Biosynthesis of secondary metabolites | 0.97 | 0.06 |
|  | Glycan biosynthesis and metabolism | 0.44 | 0.002 |
|  | Xenobiotics biodegradation and metabolism | 0.99 | 0.12 |
|  | Nucleotide metabolism | 0.16 | 0.002 |
| **PFAM groups** | Ammonium sensing | 0.85 | 0.06 |
|  | Organic N metabolism | 0.99 | 0.24 |
|  | *N permease* | 0.99 | 0.44 |
|  | *Protease* | 0.93 | 0.14 |
|  | *Peptidase* | 0.26 | 0.03 |
|  | Oxidoreductase | 0.99 | 0.27 |
|  | *Peroxidases* | 0.99 | 0.27 |
|  | *Multicopper oxidase* | 0.99 | 0.26 |
|  | Cell wall biosynthesis | 0.69 | 0.11 |
|  | *Glucan biosynthesis* | 0.93 | 0.18 |
|  | *Chitin biosynthesis* | 0.71 | 0.14 |

**Table S2**. Variance inflation factors (VIF) among the parametric model terms from four models with the most significant fungal effects. Note that none of the models had issues with high VIF.

| Predictor | VIF |
| --- | --- |
| Fungal PCoA1 | 1.916893 |
| N deposition | 2.608463 |
| Inorganic N | 1.517372 |
| MAT | 2.080684 |
| MAP | 1.322787 |
| Soil pH | 1.491643 |
| Tree type | 2.167333 |
| Gene model number | 1.300425 |
| N deposition | 2.57172 |
| Inorganic N | 2.122619 |
| MAT | 1.29585 |
| MAP | 1.540326 |
| Soil pH | 1.535454 |
| Tree type | 1.730374 |
| Energy/nutrient metabolism | 1.267954 |
| N deposition | 2.560181 |
| Inorganic N | 2.11058 |
| MAT | 1.29445 |
| MAP | 1.544625 |
| Soil pH | 1.489138 |
| Tree type | 1.741423 |
| Organic N cycling | 1.755558 |
| N deposition | 2.571185 |
| Inorganic N | 2.093616 |
| MAT | 1.292919 |
| MAP | 1.547726 |
| Soil pH | 1.508368 |
| Tree type | 2.09989 |

**Table S3**. Generalized additive modeling output summaries. Note that separate models were created for each fungal predictor. Significant fungal predictors are emphasized using bold. Splines were fit to stand age and stand density to account for non-linear correlations with tree growth. We set k = 3 (the minimum basis dimension) to reduce over-fitting. Basis dimension was also validated using the gam.check function, and specifically we validated that *p*-values were > 0.05 and k was > 1 and larger than the estimated degrees of freedom. Note that a reference model without a fungal predictor is shown immediately following the first model testing fungal composition effects. A short over-view of the sites included in the study may be found in Table S6.

|  | Estimate | SE | *t*-value | *p*-value |
| --- | --- | --- | --- | --- |
| (Intercept) | 2.45E+00 | 6.82E-01 | 3.586 | 0.000672 |
| Fungal PCoA1 | -2.95E+00 | 5.99E-01 | -4.916 | 7.10E-06 |
| N deposition | 4.43E-07 | 1.97E-04 | 0.002 | 0.99821 |
| Inorganic N | 1.45E-02 | 4.87E-02 | 0.298 | 0.766957 |
| MAT | 1.90E-02 | 3.26E-02 | 0.582 | 0.562978 |
| MAP | 1.97E-04 | 4.13E-04 | 0.476 | 0.636007 |
| Soil pH | -5.97E-03 | 9.50E-02 | -0.063 | 0.950124 |
| Tree type | -1.88E-01 | 2.32E-01 | -0.808 | 0.422422 |
|  | Est. DF | DF | *F*-value | *p*-value |
| Stand age | 1 | 1.001 | 2.359 | 0.13 |
| Stand density | 1.61 | 1.848 | 3.894 | 0.04 |
| R2-value | 0.538 |  |  |  |
| REML | 91.078 |  |  |  |
|  |  |  |  |  |
|  | Estimate | SE | *t*-value | *p*-value |
| (Intercept) | 1.3000608 | 0.7508807 | 1.731 | 0.08841 |
| N deposition | 0.0001586 | 0.0002275 | 0.697 | 0.48822 |
| Inorganic N | -0.0086298 | 0.0568286 | -0.152 | 0.8798 |
| MAT | 0.0475509 | 0.0376365 | 1.263 | 0.21121 |
| MAP | 0.000508 | 0.0004785 | 1.062 | 0.2925 |
| Soil pH | -0.0357225 | 0.1111403 | -0.321 | 0.74899 |
| Tree type | -0.726114 | 0.2401554 | -3.024 | 0.00364 |
|  | Est. DF | DF | *F*-value | *p*-value |
| Stand age | 1.001 | 1.001 | 1.785 | 0.186 |
| Stand density | 1.617 | 1.853 | 2.431 | 0.142 |
| R2-value | 0.365 |  |  |  |
| REML | 101.83 |  |  |  |
|  |  |  |  |  |
|  | Estimate | SE | *t*-value | *p*-value |
| (Intercept) | 1.2351771 | 0.8214722 | 1.504 | 0.13791 |
| Richness | 0.0021397 | 0.0157534 | 0.136 | 0.89242 |
| N deposition | 0.0001617 | 0.0002309 | 0.7 | 0.48636 |
| Inorganic N | 0.0505582 | 0.0381944 | 1.324 | 0.19061 |
| MAT | 0.0004769 | 0.0005135 | 0.929 | 0.3567 |
| MAP | -0.0093126 | 0.0616406 | -0.151 | 0.88042 |
| Soil pH | -0.0426194 | 0.1185804 | -0.359 | 0.72054 |
| Tree type | -0.6933683 | 0.2507953 | -2.765 | 0.00755 |
|  | Est. DF | DF | *F*-value | *p*-value |
| Stand age | 1.001 | 1.001 | 2.011 | 0.161 |
| Stand density | 1.864 | 2.249 | 1.969 | 0.15 |
| R2-value | 0.36 |  |  |  |
| REML | 104.98 |  |  |  |
|  |  |  |  |  |
|  | Estimate | SE | *t*-value | *p*-value |
| (Intercept) | 0.7450345 | 1.1995427 | 0.621 | 0.53689 |
| Shannon index | 0.2191733 | 0.3874643 | 0.566 | 0.57374 |
| N deposition | 0.0001561 | 0.0002268 | 0.688 | 0.49393 |
| Inorganic N | 0.051005 | 0.0381406 | 1.337 | 0.18619 |
| MAT | 0.0004073 | 0.0004981 | 0.818 | 0.41677 |
| MAP | 0.0007363 | 0.0624711 | 0.012 | 0.99064 |
| Soil pH | -0.0510309 | 0.1145026 | -0.446 | 0.65744 |
| Tree type | -0.6743936 | 0.2467397 | -2.733 | 0.00824 |
|  | Est. DF | DF | *F*-value | *p*-value |
| Stand age | 1 | 1.001 | 2.289 | 0.135 |
| Stand density | 2.186 | 2.712 | 1.745 | 0.176 |
| R2-value | 0.372 |  |  |  |
| REML | 101.5 |  |  |  |
|  |  |  |  |  |
|  | Estimate | SE | *t*-value | *p*-value |
| (Intercept) | 7.20E+00 | 1.19E+00 | 6.043 | 1.04E-07 |
| Gene models | -3.18E-04 | 5.51E-05 | -5.771 | 2.94E-07 |
| N deposition | 4.79E-05 | 1.85E-04 | 0.26 | 0.79598 |
| Inorganic N | 1.95E-02 | 3.11E-02 | 0.627 | 0.53301 |
| MAT | 6.14E-04 | 3.86E-04 | 1.588 | 0.11747 |
| MAP | -4.37E-02 | 4.64E-02 | -0.942 | 0.34979 |
| Soil pH | -1.28E-01 | 9.11E-02 | -1.401 | 0.16646 |
| Tree type | -6.63E-01 | 1.96E-01 | -3.379 | 0.00128 |
|  | Est. DF | DF | *F*-value | *p*-value |
| Stand age | 1 | 1 | 2.732 | 0.1035 |
| Stand density | 1.917 | 2.376 | 3.503 | 0.0285 |
| R2-value | 0.587 |  |  |  |
| REML | 97.042 |  |  |  |
|  |  |  |  |  |
|  | Estimate | SE | *t*-value | *p*-value |
| (Intercept) | -3.75E+00 | 1.01E+00 | -3.699 | 0.000471 |
| Energy/Nutrient metabolism | 7.12E+02 | 1.16E+02 | 6.124 | 7.58E-08 |
| N deposition | 6.76E-05 | 1.80E-04 | 0.375 | 0.708869 |
| Inorganic N | 2.05E-02 | 3.04E-02 | 0.674 | 0.502723 |
| MAT | 4.06E-04 | 3.78E-04 | 1.073 | 0.287696 |
| MAP | -4.86E-02 | 4.55E-02 | -1.068 | 0.289691 |
| Soil pH | -2.28E-02 | 8.78E-02 | -0.26 | 0.795745 |
| Tree type | -5.92E-01 | 1.93E-01 | -3.072 | 0.003192 |
|  | Est. DF | DF | *F*-value | *p*-value |
| Stand age | 1 | 1.001 | 0.442 | 0.509 |
| Stand density | 1.874 | 2.325 | 2.983 | 0.049 |
| R2-value | 0.604 |  |  |  |
| REML | 81.144 |  |  |  |
|  |  |  |  |  |
|  | Estimate | SE | *t*-value | *p*-value |
| (Intercept) | -1.36E+00 | 1.28E+00 | -1.064 | 0.29163 |
| Nitrogen metabolism | 2.10E+03 | 8.47E+02 | 2.476 | 0.01613 |
| N deposition | 1.07E-04 | 2.17E-04 | 0.493 | 0.62387 |
| Inorganic N | 6.06E-02 | 3.67E-02 | 1.651 | 0.10398 |
| MAT | 4.78E-04 | 4.54E-04 | 1.053 | 0.2964 |
| MAP | 5.70E-03 | 5.50E-02 | 0.104 | 0.91783 |
| Soil pH | -3.74E-02 | 1.06E-01 | -0.355 | 0.7241 |
| Tree type | -7.04E-01 | 2.33E-01 | -3.018 | 0.00374 |
|  | Est. DF | DF | *F*-value | *p*-value |
| Stand age | 1 | 1.001 | 0.912 | 0.343 |
| Stand density | 2.368 | 2.931 | 1.65 | 0.196 |
| R2-value | 0.43 |  |  |  |
| REML | 91.001 |  |  |  |
|  |  |  |  |  |
|  | Estimate | SE | *t*-value | *p*-value |
| (Intercept) | 2.85E+00 | 8.45E-01 | 3.378 | 0.00127 |
| Ammonium sensing | -7.23E+02 | 3.08E+02 | -2.347 | 0.02215 |
| N deposition | -6.96E-05 | 1.47E-04 | -0.472 | 0.63872 |
| Inorganic N | 5.60E-02 | 2.64E-02 | 2.121 | 0.03798 |
| MAT | 1.69E-04 | 3.45E-04 | 0.491 | 0.62547 |
| MAP | 3.44E-02 | 3.90E-02 | 0.881 | 0.38188 |
| Soil pH | 8.06E-03 | 8.19E-02 | 0.099 | 0.92186 |
| Tree type | -4.05E-01 | 1.84E-01 | -2.203 | 0.03135 |
|  | Est. DF | DF | *F*-value | *p*-value |
| Stand age | 1.967 | 1.999 | 27.02 | 1.01E-09 |
| Stand density | 1.77 | 2.194 | 1.67 | 0.179 |
| R2-value | 0.672 |  |  |  |
| REML | 77.317 |  |  |  |
|  |  |  |  |  |
|  | Estimate | SE | *t*-value | *p*-value |
| (Intercept) | 2.74E+00 | 8.67E-01 | 3.166 | 0.00242 |
| N permeases | -1.30E+03 | 4.42E+02 | -2.934 | 0.00472 |
| N deposition | 1.55E-04 | 2.15E-04 | 0.722 | 0.47285 |
| Inorganic N | 1.83E-02 | 3.73E-02 | 0.49 | 0.62573 |
| MAT | 7.17E-04 | 4.57E-04 | 1.567 | 0.1224 |
| MAP | -9.48E-03 | 5.38E-02 | -0.176 | 0.86072 |
| Soil pH | 1.21E-02 | 1.06E-01 | 0.114 | 0.90958 |
| Tree type | -7.26E-01 | 2.28E-01 | -3.188 | 0.00227 |
|  | Est. DF | DF | *F*-value | *p*-value |
| Stand age | 1.001 | 1.001 | 1.167 | 0.284 |
| Stand density | 1.54 | 1.894 | 0.936 | 0.335 |
| R2-value | 0.433 |  |  |  |
| REML | 90.692 |  |  |  |
|  |  |  |  |  |
|  | Estimate | SE | *t*-value | *p*-value |
| (Intercept) | 3.38E+00 | 1.18E+00 | 2.88 | 0.00552 |
| Proteases | -2.77E+02 | 1.22E+02 | -2.272 | 0.02666 |
| N deposition | 9.07E-05 | 2.21E-04 | 0.411 | 0.6822 |
| Inorganic N | 5.09E-02 | 3.67E-02 | 1.388 | 0.17038 |
| MAT | 4.87E-04 | 4.59E-04 | 1.062 | 0.29271 |
| MAP | -2.76E-02 | 5.52E-02 | -0.5 | 0.61861 |
| Soil pH | -1.27E-01 | 1.14E-01 | -1.112 | 0.27077 |
| Tree type | -4.95E-01 | 2.51E-01 | -1.971 | 0.05333 |
|  | Est. DF | DF | *F*-value | *p*-value |
| Stand age | 1 | 1 | 0.736 | 0.394 |
| Stand density | 2.115 | 2.624 | 2.15 | 0.114 |
| R2-value | 0.416 |  |  |  |
| REML | 93.392 |  |  |  |
|  |  |  |  |  |
|  | Estimate | SE | *t*-value | *p*-value |
| (Intercept) | 3.47E+00 | 8.41E-01 | 4.126 | 0.000115 |
| Multicopper oxidases | -6.69E+02 | 1.58E+02 | -4.233 | 8.00E-05 |
| N deposition | 2.23E-04 | 2.01E-04 | 1.108 | 0.272401 |
| Inorganic N | -1.01E-02 | 3.64E-02 | -0.276 | 0.783188 |
| MAT | 5.91E-04 | 4.23E-04 | 1.399 | 0.166822 |
| MAP | -4.76E-02 | 5.11E-02 | -0.932 | 0.35525 |
| Soil pH | 4.19E-02 | 9.98E-02 | 0.42 | 0.676172 |
| Tree type | -4.58E-01 | 2.22E-01 | -2.061 | 0.043646 |
|  | Est. DF | DF | *F*-value | *p*-value |
| Stand age | 1 | 1 | 2.249 | 0.139 |
| Stand density | 1.862 | 2.302 | 2.138 | 0.108 |
| R2-value | 0.506 |  |  |  |
| REML | 87.705 |  |  |  |
|  |  |  |  |  |
|  | Estimate | SE | *t*-value | *p*-value |
| (Intercept) | 1.11E+00 | 8.12E-01 | 1.366 | 0.1771 |
| Peroxidases | 4.15E+02 | 7.84E+02 | 0.529 | 0.5986 |
| N deposition | 1.67E-04 | 2.27E-04 | 0.735 | 0.4652 |
| Inorganic N | 5.04E-02 | 3.81E-02 | 1.321 | 0.1915 |
| MAT | 4.62E-04 | 4.80E-04 | 0.964 | 0.3389 |
| MAP | -1.64E-02 | 5.72E-02 | -0.286 | 0.776 |
| Soil pH | -3.48E-02 | 1.11E-01 | -0.314 | 0.7548 |
| Tree type | -6.44E-01 | 2.63E-01 | -2.446 | 0.0174 |
|  | Est. DF | DF | *F*-value | *p*-value |
| Stand age | 1 | 1 | 1.49 | 0.227 |
| Stand density | 2.173 | 2.696 | 1.782 | 0.17 |
| R2-value | 0.371 |  |  |  |
| REML | 93.905 |  |  |  |
|  |  |  |  |  |
|  | Estimate | SE | *t*-value | *p*-value |
| (Intercept) | 5.54E+00 | 1.43E+00 | 3.871 | 0.00027 |
| Organic N cycling | -1.96E+02 | 5.77E+01 | -3.398 | 0.00121 |
| N deposition | 8.80E-05 | 2.10E-04 | 0.419 | 0.67686 |
| Inorganic N | 3.73E-02 | 3.52E-02 | 1.058 | 0.29413 |
| MAT | 5.13E-04 | 4.39E-04 | 1.167 | 0.24767 |
| MAP | -3.61E-02 | 5.29E-02 | -0.682 | 0.49772 |
| Soil pH | -7.46E-02 | 1.03E-01 | -0.726 | 0.47072 |
| Tree type | -3.57E-01 | 2.46E-01 | -1.45 | 0.15213 |
|  | Est. DF | DF | *F*-value | *p*-value |
| Stand age | 1.001 | 1.001 | 0.462 | 0.499 |
| Stand density | 2.002 | 2.484 | 2.182 | 0.107 |
| R2-value | 0.465 |  |  |  |
| REML | 91.299 |  |  |  |
|  |  |  |  |  |
|  | Estimate | SE | *t*-value | *p*-value |
| (Intercept) | 2.13E-01 | 9.89E-01 | 0.215 | 0.83066 |
| Glycan biosynthesis | 1.42E+02 | 8.82E+01 | 1.607 | 0.11339 |
| N deposition | 1.40E-04 | 2.23E-04 | 0.63 | 0.53127 |
| Inorganic N | 4.31E-02 | 3.77E-02 | 1.143 | 0.2578 |
| MAT | 4.54E-04 | 4.68E-04 | 0.97 | 0.33581 |
| MAP | 7.26E-03 | 5.75E-02 | 0.126 | 0.89986 |
| Soil pH | -6.09E-02 | 1.10E-01 | -0.554 | 0.58194 |
| Tree type | -7.26E-01 | 2.40E-01 | -3.026 | 0.00365 |
|  | Est. DF | DF | *F*-value | *p*-value |
| Stand age | 1 | 1 | 1.368 | 0.247 |
| Stand density | 2.189 | 2.713 | 1.639 | 0.205 |
| R2-value | 0.394 |  |  |  |
| REML | 94.946 |  |  |  |
|  |  |  |  |  |
|  | Estimate | SE | *t*-value | *p*-value |
| (Intercept) | 1.13E+00 | 8.19E-01 | 1.379 | 0.1731 |
| Glucan biosynthesis | 3.61E+02 | 8.37E+02 | 0.432 | 0.6675 |
| N deposition | 1.49E-04 | 2.28E-04 | 0.653 | 0.5161 |
| Inorganic N | 5.18E-02 | 3.83E-02 | 1.351 | 0.1816 |
| MAT | 4.73E-04 | 4.77E-04 | 0.99 | 0.326 |
| MAP | -1.56E-02 | 5.71E-02 | -0.274 | 0.7849 |
| Soil pH | -4.60E-02 | 1.14E-01 | -0.403 | 0.6881 |
| Tree type | -6.45E-01 | 2.69E-01 | -2.399 | 0.0196 |
|  | Est. DF | DF | *F*-value | *p*-value |
| Stand age | 1 | 1 | 1.552 | 0.218 |
| Stand density | 2.27 | 2.814 | 1.794 | 0.165 |
| R2-value | 0.372 |  |  |  |
| REML | 93.885 |  |  |  |
|  |  |  |  |  |
|  | Estimate | SE | *t*-value | *p*-value |
| (Intercept) | 2.05E+00 | 8.74E-01 | 2.339 | 0.02272 |
| Chitin biosynthesis | -4.63E+02 | 2.84E+02 | -1.628 | 0.10884 |
| N deposition | 8.17E-05 | 2.28E-04 | 0.359 | 0.72097 |
| Inorganic N | 7.33E-02 | 4.00E-02 | 1.831 | 0.07209 |
| MAT | 5.10E-04 | 4.68E-04 | 1.09 | 0.2802 |
| MAP | -2.11E-02 | 5.61E-02 | -0.376 | 0.70851 |
| Soil pH | -5.96E-02 | 1.10E-01 | -0.543 | 0.58935 |
| Tree type | -6.56E-01 | 2.40E-01 | -2.729 | 0.00832 |
|  | Est. DF | DF | *F*-value | *p*-value |
| Stand age | 1.001 | 1.002 | 0.833 | 0.365 |
| Stand density | 2.134 | 2.651 | 1.59 | 0.211 |
| R2-value | 0.394 |  |  |  |
| REML | 93.746 |  |  |  |
|  |  |  |  |  |
|  | Estimate | SE | *t*-value | *p*-value |
| (Intercept) | -8.94E-01 | 9.59E-01 | -0.932 | 0.354877 |
| Carbohydrate metabolism | 9.60E+01 | 2.93E+01 | 3.275 | 0.001757 |
| N deposition | 2.66E-05 | 2.14E-04 | 0.124 | 0.901361 |
| Inorganic N | 4.60E-02 | 3.53E-02 | 1.304 | 0.19713 |
| MAT | 4.36E-04 | 4.41E-04 | 0.989 | 0.326573 |
| MAP | 1.05E-02 | 5.32E-02 | 0.197 | 0.844848 |
| Soil pH | -7.21E-02 | 1.03E-01 | -0.699 | 0.487077 |
| Tree type | -8.08E-01 | 2.28E-01 | -3.552 | 0.000752 |
|  | Est. DF | DF | *F*-value | *p*-value |
| Stand age | 1 | 1 | 0.503 | 0.481 |
| Stand density | 2.131 | 2.643 | 1.659 | 0.202 |
| R2-value | 0.462 |  |  |  |
| REML | 92.314 |  |  |  |
|  |  |  |  |  |
|  | Estimate | SE | *t*-value | *p*-value |
| (Intercept) | -8.54E-01 | 1.11E+00 | -0.769 | 0.44487 |
| Lipid metabolism | 1.09E+02 | 4.35E+01 | 2.503 | 0.01508 |
| N deposition | 5.62E-05 | 2.20E-04 | 0.256 | 0.79915 |
| Inorganic N | 5.30E-02 | 3.64E-02 | 1.454 | 0.15126 |
| MAT | 4.22E-04 | 4.55E-04 | 0.928 | 0.35701 |
| MAP | 2.15E-03 | 5.48E-02 | 0.039 | 0.96885 |
| Soil pH | -6.54E-02 | 1.06E-01 | -0.615 | 0.54108 |
| Tree type | -8.03E-01 | 2.37E-01 | -3.389 | 0.00125 |
|  | Est. DF | DF | *F*-value | *p*-value |
| Stand age | 1 | 1 | 0.761 | 0.386 |
| Stand density | 2.272 | 2.814 | 1.562 | 0.224 |
| R2-value | 0.429 |  |  |  |
| REML | 93.906 |  |  |  |
|  |  |  |  |  |
|  | Estimate | SE | *t*-value | *p*-value |
| (Intercept) | -3.54E+00 | 1.17E+00 | -3.03 | 0.003609 |
| AA metabolism | 2.06E+02 | 4.18E+01 | 4.923 | 7.00E-06 |
| N deposition | -4.87E-05 | 1.98E-04 | -0.246 | 0.806197 |
| Inorganic N | 4.92E-02 | 3.23E-02 | 1.522 | 0.133209 |
| MAT | 4.47E-04 | 4.06E-04 | 1.102 | 0.27507 |
| MAP | -1.12E-02 | 4.84E-02 | -0.231 | 0.818023 |
| Soil pH | -9.33E-02 | 9.50E-02 | -0.982 | 0.3301 |
| Tree type | -8.22E-01 | 2.08E-01 | -3.96 | 0.000202 |
|  | Est. DF | DF | *F*-value | *p*-value |
| Stand age | 1 | 1 | 0.158 | 0.692 |
| Stand density | 1.971 | 2.447 | 1.826 | 0.166 |
| R2-value | 0.544 |  |  |  |
| REML | 86.631 |  |  |  |

**Table S4.** Summary of the indicator species analysis.

|  | **Tree** | **Slow** | **Fast** | **Ind Val.** | ***p*-value** | **Species/taxonomy** |
| --- | --- | --- | --- | --- | --- | --- |
| **KF007260** | Needle | 1 | 0 | 0.83666 | 0.001 | Atheliaceae |
| **EU427330** | Needle | 1 | 0 | 0.828079 | 0.007 | Thelephoraceae |
| **UDB000436** | Needle | 1 | 0 | 0.730897 | 0.039 | Imleria badia (SH216653.07FU) |
| **JN006469** | Needle | 1 | 0 | 0.707107 | 0.022 | Archaeorhizomyces |
| **FJ807983** | Needle | 1 | 0 | 0.707107 | 0.01 | Atheliaceae |
| **UDB003332** | Needle | 1 | 0 | 0.632456 | 0.044 | Lactifluus vellereus (SH190732.07FU) |
| **HQ625478** | Needle | 1 | 0 | 0.632456 | 0.041 | Agaricomycetes |
| **FN610860** | Needle | 0 | 1 | 0.865094 | 0.001 | Cenococcum |
| **AB254392** | Needle | 0 | 1 | 0.85485 | 0.001 | Atheliaceae |
| **UDB001740** | Needle | 0 | 1 | 0.848528 | 0.002 | Piloderma fallax (SH203892.07FU) |
| **AY394919** | Needle | 0 | 1 | 0.779036 | 0.009 | Cenococcum geophilum (SH199612.07FU) |
| **DQ097870** | Needle | 0 | 1 | 0.774597 | 0.007 | Cortinariaceae |
| **KC581331** | Needle | 0 | 1 | 0.774597 | 0.005 | Russulaceae |
| **UDB001726** | Needle | 0 | 1 | 0.774597 | 0.008 | Piloderma |
| **UDB001739** | Needle | 0 | 1 | 0.774597 | 0.005 | Piloderma byssinum (SH208786.07FU) |
| **FJ378819** | Needle | 0 | 1 | 0.761124 | 0.005 | Lactarius |
| **EU057126** | Needle | 0 | 1 | 0.740153 | 0.042 | Atheliaceae |
| **UDB016009** | Needle | 0 | 1 | 0.731529 | 0.036 | Russula ochroleuca (SH190469.07FU) |
| **FJ236853** | Needle | 0 | 1 | 0.632456 | 0.037 | Tomentella |
| **UDB002249** | Needle | 0 | 1 | 0.632456 | 0.04 | Cortinariaceae |
| **DQ061887** | Needle | 0 | 1 | 0.632456 | 0.034 | Russulaceae |
| **UDB017899** | Needle | 0 | 1 | 0.632456 | 0.042 | Lactifluus glaucescens (SH214427.07FU) |
| **FJ807967** | Needle | 0 | 1 | 0.632456 | 0.04 | Thelephoraceae |
| **FJ039708** | Needle | 0 | 1 | 0.632456 | 0.033 | Cortinariaceae |
| **UDB016038** | Needle | 0 | 1 | 0.632456 | 0.04 | Russula griseascens (SH218422.07FU) |
| **KF717578** | Needle | 0 | 1 | 0.632456 | 0.04 | Otidea leporina (SH218038.07FU) |
| **UDB011572** | Needle | 0 | 1 | 0.632456 | 0.037 | Tricholoma inamoenum (SH190400.07FU) |
| **UDB000340** | Broad | 1 | 0 | 0.960769 | 0.031 | Russulaceae |
| **UDB011494** | Broad | 1 | 0 | 1 | 0.046 | Lactarius quietus (SH220118.07FU) |
| **DQ990846** | Broad | 1 | 0 | 1 | 0.046 | Russulaceae |
| **FM205502** | Broad | 1 | 0 | 1 | 0.046 | Tuber borchii (SH216304.07FU) |
| **UDB001404** | Broad | 0 | 1 | 1 | 0.003 | Xerocomellus pruinatus (SH197705.07FU) |
| **UDB017328** | Broad | 0 | 1 | 0.923936 | 0.007 | Elaphomycetaceae |
| **UDB016009** | Broad | 0 | 1 | 0.885061 | 0.039 | Russula ochroleuca (SH190469.07FU) |
| **AM087246** | Broad | 0 | 1 | 1 | 0.044 | Hydnum |
| **UDB005288** | Broad | 0 | 1 | 1 | 0.044 | Cantharellaceae |
| **FR750607** | Broad | 0 | 1 | 1 | 0.044 | Agaricomycetes |
| **FJ440882** | Broad | 0 | 1 | 1 | 0.044 | Cenococcum |
| **JF519061** | Broad | 0 | 1 | 1 | 0.044 | Thelephoraceae |
| **GQ219881** | Broad | 0 | 1 | 1 | 0.044 | Agaricomycetes |

**Table S5.** The number of ectomycorrhizal OTUs and proportion of sequences assigned to exploration types from the Fungal Traits database (v 1.2). All assignments were made at the genus level. Only 59% of the OTUs were assigned genus-level taxonomic annotations.

|  | **Proportion of OTUs** | **Proportion of sequences** |
| --- | --- | --- |
| Total assignments | 38% | 32% |

**Table S6.** A basic description of the ICP plots included in the full statistical models. Only this sub-sample of the plots had information on all of the co-variables used in the full statistical models.

| **ICP country** | | **ICP plot** | **Average stand age** | | | **Tree type** | |
| --- | --- | --- | --- | --- | --- | --- | --- |
| 1 | 37 | | | <= 20 | Needleleaf | |  |
| 1 | 41 | | | <= 20 | Needleleaf | |  |
| 1 | 46 | | | <= 20 | Needleleaf | |  |
| 1 | 57 | | | <= 20 | Broadleaf | |  |
| 1 | 63 | | | <= 20 | Broadleaf | |  |
| 11 | 115 | | | 61-80 | Broadleaf | |  |
| 11 | 30 | | | 81-100 | Needleleaf | |  |
| 11 | 5 | | | 81-100 | Needleleaf | |  |
| 15 | 10 | | | 81-100 | Needleleaf | |  |
| 15 | 12 | | | 61-80 | Needleleaf | |  |
| 15 | 13 | | | 61-80 | Needleleaf | |  |
| 15 | 16 | | | 61-80 | Needleleaf | |  |
| 15 | 17 | | | 61-80 | Needleleaf | |  |
| 15 | 19 | | | >120 | Needleleaf | |  |
| 15 | 20 | | | >120 | Needleleaf | |  |
| 15 | 3 | | | >120 | Needleleaf | |  |
| 15 | 5 | | | 61-80 | Needleleaf | |  |
| 15 | 6 | | | 41-60 | Needleleaf | |  |
| 2 | 1 | | | 61-80 | Needleleaf | |  |
| 2 | 9 | | | >120 | Broadleaf | |  |
| 4 | 101 | | | 101-120 | Broadleaf | |  |
| 4 | 1201 | | | 81-100 | Needleleaf | |  |
| 4 | 1205 | | | 81-100 | Needleleaf | |  |
| 4 | 1401 | | | 81-100 | Needleleaf | |  |
| 4 | 1402 | | | 101-120 | Needleleaf | |  |
| 4 | 1405 | | | 101-120 | Needleleaf | |  |
| 4 | 301 | | | >120 | Broadleaf | |  |
| 4 | 304 | | | >120 | Broadleaf | |  |
| 4 | 305 | | | 101-120 | Needleleaf | |  |
| 4 | 306 | | | >120 | Broadleaf | |  |
| 4 | 307 | | | 61-80 | Needleleaf | |  |
| 4 | 502 | | | >120 | Broadleaf | |  |
| 4 | 503 | | | >120 | Broadleaf | |  |
| 4 | 606 | | | >120 | Broadleaf | |  |
| 4 | 608 | | | >120 | Broadleaf | |  |
| 4 | 705 | | | >120 | Broadleaf | |  |
| 4 | 706 | | | 101-120 | Broadleaf | |  |
| 4 | 901 | | | 81-100 | Needleleaf | |  |
| 4 | 906 | | | 61-80 | Needleleaf | |  |
| 4 | 908 | | | 61-80 | Needleleaf | |  |
| 4 | 921 | | | 81-100 | Broadleaf | |  |
| 5 | 1 | | | >120 | Broadleaf | |  |
| 5 | 10 | | | 81-100 | Needleleaf | |  |
| 5 | 12 | | | 61-80 | Broadleaf | |  |
| 5 | 20 | | | >120 | Broadleaf | |  |
| 5 | 3 | | | >120 | Broadleaf | |  |
| 50 | 15 | | | 81-100 | Needleleaf | |  |
| 50 | 18 | | | 101-120 | Needleleaf | |  |
| 50 | 2 | | | >120 | Needleleaf | |  |
| 50 | 3 | | | >120 | Broadleaf | |  |
| 52 | 5 | | | 41-60 | Broadleaf | |  |
| 53 | 116 | | | 61-80 | Broadleaf | |  |
| 53 | 206 | | | 101-120 | Needleleaf | |  |
| 53 | 212 | | | 61-80 | Needleleaf | |  |
| 53 | 405 | | | 61-80 | Needleleaf | |  |
| 53 | 513 | | | 81-100 | Needleleaf | |  |
| 53 | 804 | | | 61-80 | Broadleaf | |  |
| 53 | 816 | | | 61-80 | Needleleaf | |  |
| 58 | 2015 | | | >120 | Broadleaf | |  |
| 58 | 2061 | | | 81-100 | Needleleaf | |  |
| 58 | 2103 | | | 101-120 | Broadleaf | |  |
| 58 | 2161 | | | 101-120 | Needleleaf | |  |
| 58 | 2401 | | | 81-100 | Needleleaf | |  |
| 59 | 2 | | | 81-100 | Needleleaf | |  |
| 59 | 3 | | | 61-80 | Needleleaf | |  |
| 59 | 7 | | | 81-100 | Needleleaf | |  |
| 59 | 8 | | | 41-60 | Needleleaf | |  |
| 59 | 9 | | | 41-60 | Needleleaf | |  |
| 6 | 512 | | | 61-80 | Broadleaf | |  |
| 6 | 715 | | | 41-60 | Needleleaf | |  |
| 60 | 4 | | | 81-100 | Needleleaf | |  |

**Fig. S1.** **Decision tree and detailed methodology of fungal functional gene assignments.** This decision tree outlines the assignment of functional gene numbers to ITS sequences based on direct matching between species and reference genomes in the MycoCosm database and assignments at the genus-level based on phylogenetic signal.


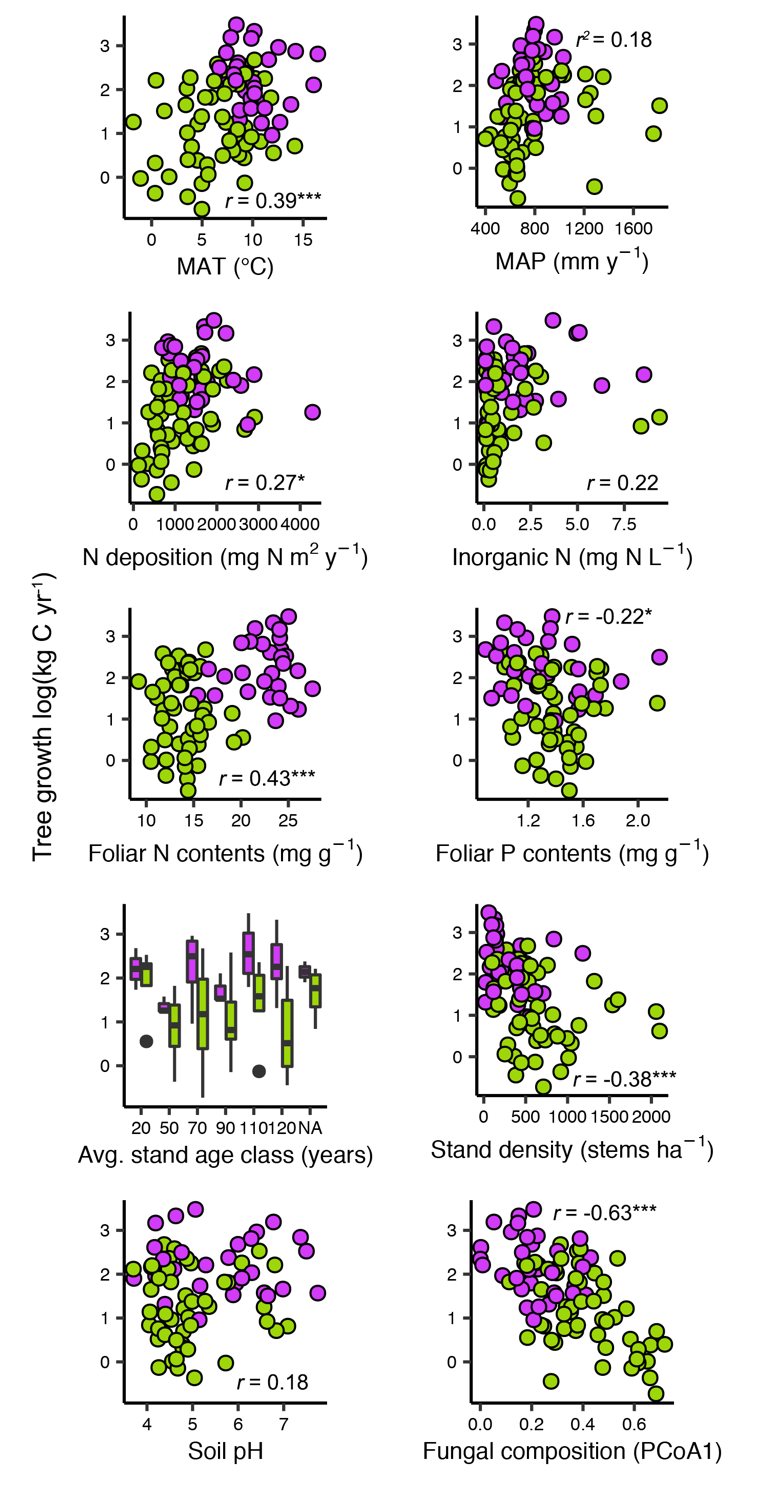


**Fig. S2**. **Correlations between tree growth and the predictor variables included in our analysis.** Values show pearson correlations and significant differences are indicated using asterisks where * = *p* < 0.05, ** = *p* < 0.01, *** = *p* < 0.001. Needleleaf sites are in green while broadleaf sites are in purple. Note that there were too many fungal predictors to visualize here, so we only show fungal community composition. Note that we also included foliar N and foliar P contents in the analysis; however, these were ultimately not included in the statistical models because foliar N contents was not independent from tree type (broad vs. needleleaf) nor was it correlated with tree growth when examined separately by tree type. Foliar P contents was also not strongly correlated with tree growth rate, and it was not correlated when examined separately by tree type, so it was not included in the analysis

**Fig. S3. Variance partitioning of the deviance explained in tree growth rate by predictor variables in the fungal community composition generalized additive growth model.** Variance explained was computed as the difference in deviance between alternative models fit without each model term and the full model divided by deviance explained when the independent variables are zero or have no influence. A simplified example with two predictors is shown below:

m1 = gam(y ~ x1 + x2)

m2 = gam(y ~ x1)

m3 = gam(y ~ x2)

m0 = gam(y ~ 1)

Proportion of variance explained by x2 = (deviance(m2) – deviance(m1))/deviance(m0).

**Fig. S4. Correlations between tree growth rate and fungal community composition separated by tree species**. Values show predicted tree growth while controlling for the influence of other covariates in the full statistical model.

**Fig. S5.** **The correlation between fungal organic N cycling gene proportions and inorganic N availability.** The correlation weakly significant (*R*^2^ = 0.07, *p* = 0.03).

**Fig. 6. Correlation between tree growth rate and the relative abundance of contact type EMF.** Values show predicted tree growth while controlling for the influence of other covariates in the full statistical model. This correlation is weakly significant (*R*^2^ = 0.11, *p* < 0.001).

**Fig. S7.** **The relative abundance of medium-distance fringe EMF across different forest age classes.** The ‘NA’ bar shows the proportion of medium-distance fringe (%) EMF at sites for which there was EMF community composition information but no details on forest stand age. These forests were not included in the full statistical tree growth models.
